# Activating PIK3CA mutation promotes adipogenesis of adipose-derived stem cells in macrodactyly via up-regulation of E2F1

**DOI:** 10.1038/s41419-020-02806-1

**Published:** 2020-07-30

**Authors:** Bin Sun, Yongkang Jiang, Hengqing Cui, Xia Fang, Gang Han, Xinyi Dai, Shengbo Zhou, Hailei Mao, Bin Wang

**Affiliations:** 1 Department of Plastic and Reconstructive Surgery, Shanghai Ninth People’s Hospital, Shanghai Jiao Tong University School of Medicine, Shanghai, China; 2 Department of Anesthesiology and Critical Care Medicine, Zhongshan Hospital, Fudan University, 200032 Shanghai, China

**Keywords:** Lipids, Stem-cell research

## Abstract

Macrodactyly is a congenital malformation characterized by enlargement of bone and soft tissues in limbs, typically with excessive accumulation of adipose tissues. Although gain-of-function mutation of PIK3CA has been identified in macrodactyly, the mechanism of PIK3CA mutation in adipose accumulation is poorly understood. In this study, we found that adipocytes from macrodactyly were more hypertrophic than those observed in polydactyly. PIK3CA (H1047R) activating mutation and enhanced activity of PI3K/AKT pathway were detected in macrodactylous adipose-derived stem cells (Mac-ADSCs). Compared to polydactyly-derived ADSCs (Pol-ADSCs), Mac-ADSCs had higher potential in adipogenic differentiation. Knockdown of PIK3CA or inhibition by BYL-719, a potent inhibitor of PIK3CA, impaired adipogenesis of Mac-ADSCs in vitro. In vivo study, either transient treatment of ADSCs or intragastrical gavage with BYL-719 inhibited the adipose formation in patient-derived xenograft (PDX). Furthermore, RNA-seq revealed that E2F1 was up-regulated in Mac-ADSCs and its knockdown blocked the PIK3CA-promoted adipogenesis. Our findings demonstrated that PIK3CA activating mutation promoted adipogenesis of ADSCs in macrodactyly, and that this effect was exerted by the up-regulation of E2F1. This study revealed a possible mechanism for adipose accumulation in macrodactyly and suggested BYL-719 as a potential therapeutic agent for macrodactyly treatment.

## Introduction

Macrodactyly is a rare nonhereditary congenital disorder characterized by enlargement of bone and soft tissues in digits or limbs, which is considered as a benign tumor-like condition^1^. Its incidence is estimated to be 1:18,000^2,3^. Despite of a relatively low prevalence, a large number of patients still suffer from this disease in China owing to the huge population of 1.4 billion. Our team treats more than 100 patients with macrodactyly annually. The grossly deformed and giant digits often impose a profound negative impact on patients and their families both economically and psychologically. Although a number of surgical options have been proposed over the past 50 years, none have been able to achieve satisfactory esthetic and functional results^4^. Debulking is often the choice for mild macrodactyly, while concerns exist over surgical reduction due to the high chance of recurrence. For progressive macrodactyly, amputation is the primary and last treatment option^5,6^. Thus, there is an urgent unmet need to develop novel and effective therapies. However, the unclear molecular mechanism of macrodactyly hinders this effort.

Aggressive overgrowth of adipose tissue is a typical feature of macrodactyly. Previous study of our team has also shown that there was excessive accumulation of adipose in macrodactyly^7^. Adipogenesis is the process during which preadipocytes developed into mature adipocytes and this transition is regulated by complex array of transcriptional factors and related signals pathways like PI3K/AKT pathway^8^. Recent studies demonstrated that increased adipogenesis could lead to adipose tissue accumulation in obesity and lipomas^9,10^.

Somatic mutation of PIK3CA has been identified in macrodactyly^11,12^. PIK3CA encodes the 110-kD catalytic α-subunit (p110α) of Class I PI3K, which is a master regulator of cell survival and metabolism. PI3K controls various cellular processes via PI3K/AKT pathway^13,14^ and this pathway is aberrantly activated in almost 70% of solid tumors^15^. Studies have revealed that PI3K/AKT signaling regulates the differentiation of adipose precursor cells^16^ and the progress of adipogenesis through translocation of downstream transcription factors^17^. However, no study has investigated the impact of PIK3CA activating mutation on adipogenesis in macrodactyly and its underlying molecular mechanism.

In the present study, we found that PIK3CA activating mutation promoted adipogenesis of Mac-ADSCs in macrodactyly, and that this effect was exerted by the up-regulation of E2F1. This study illustrated a possible mechanism for adipose accumulation in macrodactyly and indicated BYL-719 as a potential therapeutic agent for macrodactyly treatment.

## Results

### Excessive adipose accumulation and adipocyte hypertrophy in macrodactyly

**Fig. 1:**
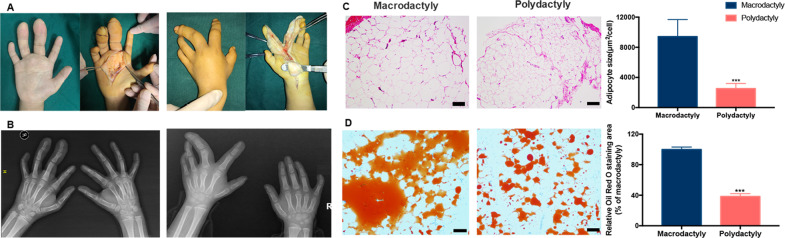
Excessive accumulation of adipose tissue and hypertrophy of adipocytes in macrodactyly. **a** Excessive accumulation of adipose tissue was observed during debulking surgery in two patients with finger macrodactyly (one left hand and the other right). **b** Radiograph of these two patients with finger macrodactyly. **c**, **d** H&E and Oil Red O staining of adipose tissue of polydactyly and macrodactyly. The adipocyte size and Oil Red O staining area were measured and quantified by using Image-Pro software. ***p < 0.001 by unpaired *t* test. Scale bar: 100 μm.

Increased soft tissue volume with especially the accumulation of adipose tissue was observed in macrodactyly during debulking surgery (Fig. 1a). Enlarged phalanges were also observed in radiographic examination (Fig. 1b). To understand the pathological changes in macrodactyly, adipose tissue of macrodactyly and polydactyly was stained with H&E and Oil Red O staining. The size of adipocytes in macrodactyly were about 3.5 times larger than those in polydactyly (*p* < 0.05), indicating that macrodactylous adipocytes were significantly more hypertrophic (Fig. 1c). Lipid accumulation was also more excessive in macrodactyly compared to polydactyly (Fig. 1d).

### Gain-of-function mutation of PIK3CA in Mac-ADSCs

**Fig. 2:**
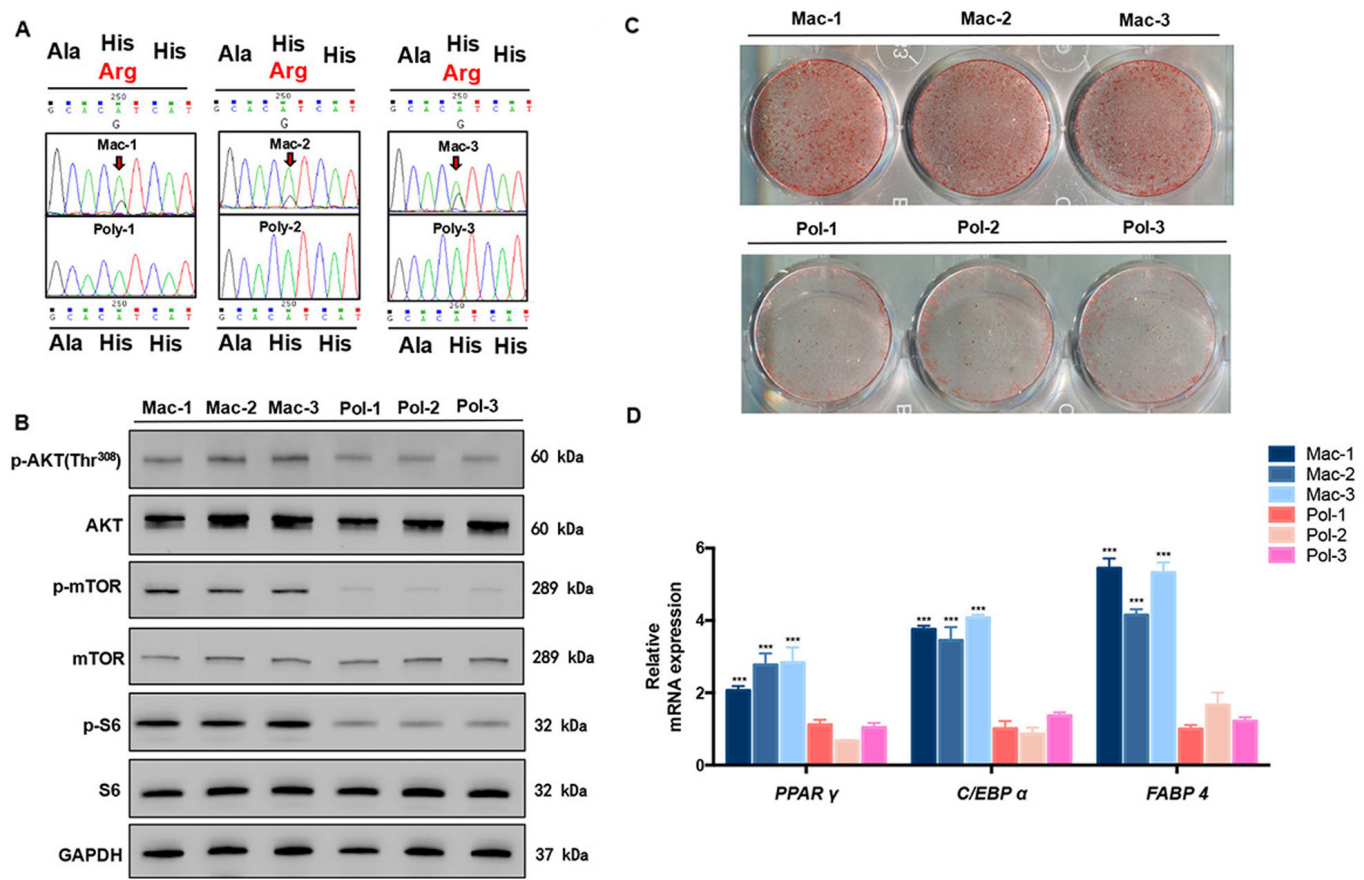
Increased adipogenesis in Mac-ADSCs with gain-of-function of PIK3CA. **a** Sanger sequencing was used to detect mutations in Mac-ADSCs and Pol-ADSCs. **b** The expression of proteins downstream of PI3K/AKT pathway in Mac-ADSCs and Pol-ADSCs was detected by western blotting. GAPDH was used as a loading control. **c** The Oil Red O staining of Mac-ADSCs and Pol-ADSCs was conducted after adipogenic induction for 15 days (3 macrodactyly patients vs 3 polydactyly patients). **d** The mRNA levels of adipogenic markers *PPAR γ*, *C/EBP α*, and *FABP4* in Mac-ADSCs and Pol-ADSCs were detected by RT-qPCR after adipogenic induction for 3 days (3 macrodactyly patients vs 3 polydactyly patients). Data are shown as the mean ± SD from three independent experiments. ***p < 0.001 by unpaired *t* test.

Sanger sequencing was conducted to assess PIK3CA mutation in Mac-ADSCs and Pol-ADSCs. Notably, PIK3CA mutation (H1047R) were detected in all Mac-ADSCs but not in any Pol-ADSCs (Fig. 2a). Western blotting revealed that the expression of phosphorylated AKT, mTOR and S6 was increased in all Mac-ADSCs, indicating enhanced activity of PI3K/AKT pathway (Fig. 2b).

### Increased potential of adipogenic differentiation in Mac-ADSCs

Adipogenesis is one of the critical steps in the development of adipose tissue^18^. To explore whether ADSCs play a role in excessive accumulation of adipose in macrodactyly, we evaluated the adipogenesis potential of cultured Mac-ADSCs and Pol-ADSCs. Mac- and Pol-ADSCs showed similar morphology in culture and expressed identical ADSCs markers (Fig. S1). After adipogenic induction, Mac-ADSCs exhibited remarkably more Oil Red O stained cells as compared to Pol-ADSCs (Fig. 2c). Consistently, the expression of adipogenic markers such as *PPAR γ*, *C/EBP α*, and *FABP4*^19^ was higher in Mac-ADSCs (*p* < 0.001; Fig. 2d).

### PIK3CA-knockdown impaired adipogenesis of Mac-ADSCs

**Fig. 3:**
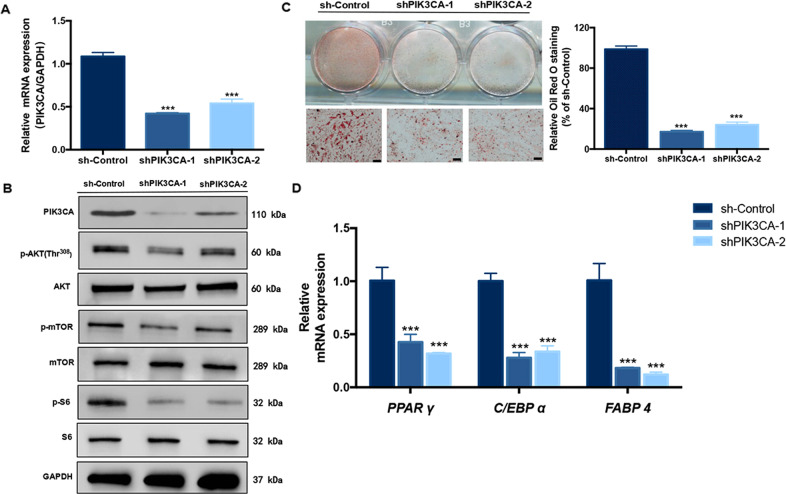
Knockdown of PIK3CA impaired adipogenic differentiation of Mac-ADSCs. **a** RT-qPCR verified the silencing of PIK3CA after transfection of the shRNA into Mac-ADSCs. **b** The levels of proteins downstream of PI3K/AKT pathway in PIK3CA-knockdown Mac-ADSCs were determined by western blotting. GAPDH was used as a loading control. **c** Oil Red O staining and quantitative assessment of sh-Control, shPIK3CA-1 and shPIK3CA-2 were conducted after adipogenic induction for 15 days. Scale bar: 50 μm. **d** The mRNA levels of *PPAR γ*, *C/EBP α,* and *FABP4* in PIK3CA-knockdown Mac-ADSCs were measured by RT-qPCR on Day 3 of adipogenic differentiation. Data shown here are mean ± SD from three independent experiments. ***p < 0.001 by ANOVA analysis.

To understand whether the increased adipogenic potential originated from activation of PIK3CA, we knocked down PIK3CA using lentiviral shRNAs (shPIK3CA). RT-qPCR showed that the expression of PIK3CA declined by ~50% after infection (Fig. 3a). Knockdown of PIK3CA also resulted in a notable decrease in protein expression of PIK3CA and proteins downstream of PI3K/AKT pathway, such as phosphorylated AKT, mTOR, and S6 (Fig. 3b). Meanwhile, the mRNA levels of the lipogenic genes *PPAR γ*, *C/EBP α*, and *FABP4* significantly decreased in response to the knockdown of PIK3CA (Fig. 3d). Moreover, consistent with the above observations, Oil Red O staining showed that area of positive staining decreased significantly by PIK3CA repression (Fig. 3c).

### PIK3CA inhibitor BYL-719 attenuated adipogenesis of Mac-ADSCs

**Fig. 4:**
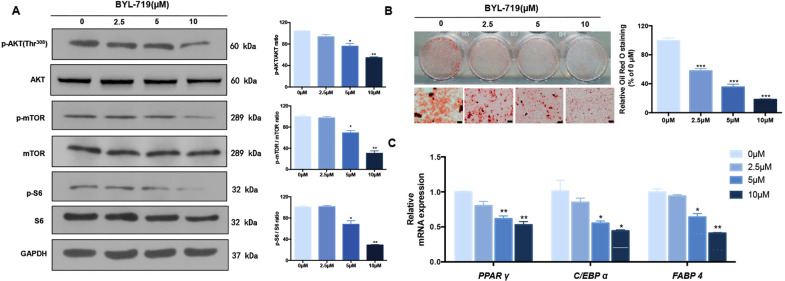
BYL-719 attenuated adipogenic differentiation of Mac-ADSCs in a dose-dependent manner. **a** The phosphorylation levels of AKT, mTOR, and S6 were determined by western blotting in Mac-ADSCs after BYL-719 treatment for 6 h. GAPDH was used as a loading control (left). Band density was quantitated by Image-Pro software (right). **b** The mature adipocytes with lipid droplets were visualized by Oil Red O staining on day 15 after BYL-719 treatment. Quantitative assessment of Oil Red O staining was shown in right. **c** The expression of adipogenic marker genes *PPAR γ*, *C/EBP α*, and *FABP4* was determined by RT-qPCR in BYL-719 treated Mac-ADSCs on day 3 of adipogenic differentiation. Data shown here are mean ± SD from three independent experiments. *p < 0.05, **p < 0.01, ***p < 0.001 by ANOVA analysis. Scale bar: 50 μm.

To examine whether inhibition of PIK3CA also attenuates adipogenesis, we treated Mac-ADSCs with PIK3CA specific inhibitor BYL-719. Phosphorylation level of AKT, mTOR and S6 was suppressed in a dose-dependent manner (Fig. 4a). Consistently, we observed that lipid accumulation of Mac-ADSCs after adipogenic induction was reduced by BYL-719 treatment (Fig. 4b). The area of positive adipocyte staining was reduced to 20.2 ± 2% following the treatment of 10 μM BYL-719. Further, the mRNA expression of *PPAR γ*, *C/EBP α*, and *FABP4* was also down-regulated dose-dependently (Fig. 4c).

### BYL-719 suppressed adipogenesis in PDX model

**Fig. 5:**
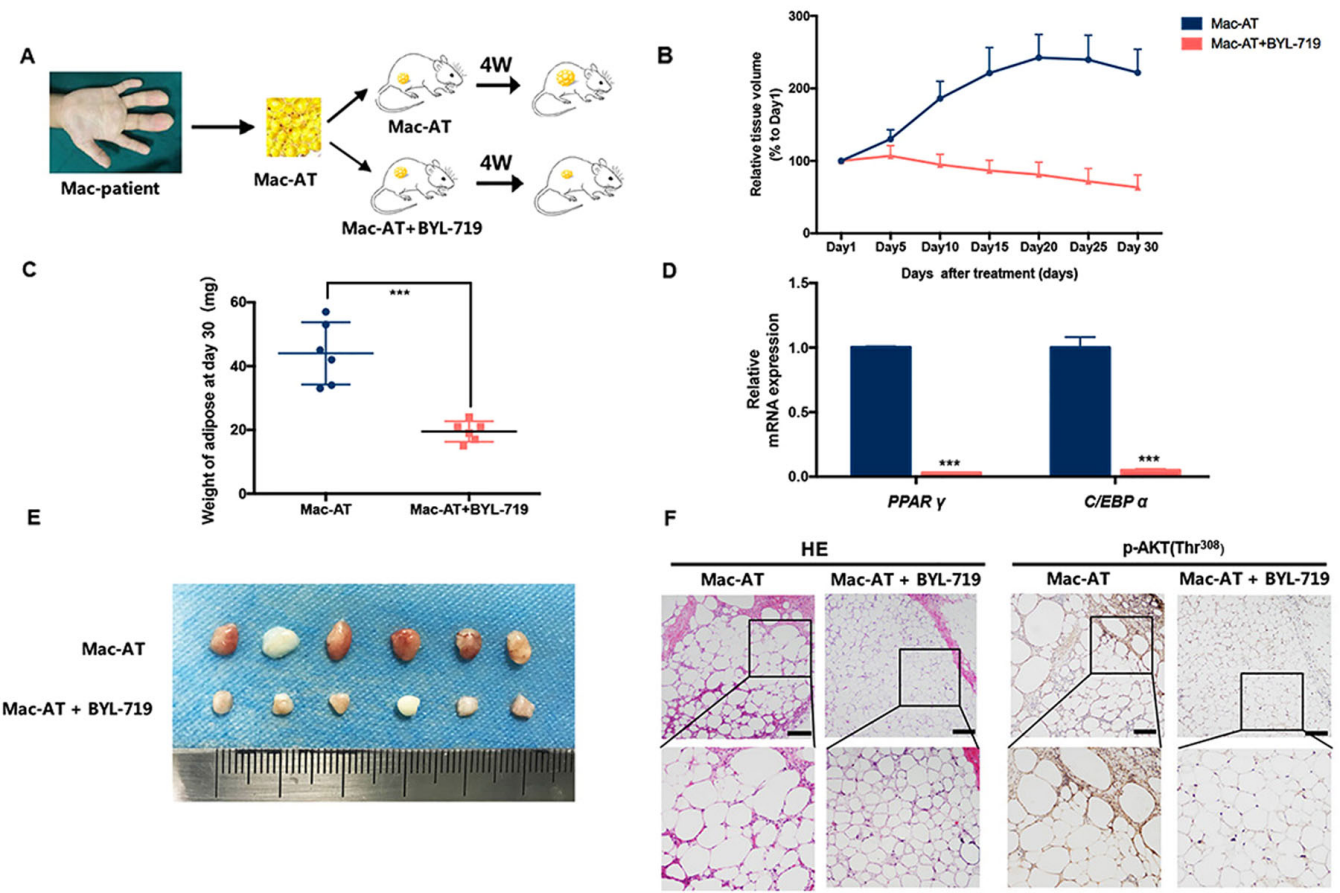
BYL-719 inhibited adipose formation in vivo. **a** Schematic description of the experimental procedure for PDX model. **b** Relative tissue volume curves of macrodactyly xenografts from treated with BYL-719 or control for 30 days (*n* = 6). **c** Weight of BYL-719 treated and control xenografts (*n* = 6). **d** The mRNA expression levels of *PPAR γ and C/EBP α* of BYL-719 treated or control Mac-AT were determined by RT-qPCR on day 30. **e**. Image of macrodactyly xenografts from PDX model after 30 days’ implantation (*n* = 6). **f** H&E staining of macrodactyly xenografts treated with BYL-719 or control on day 30 (left). Immunohistochemical staining for p-AKT of macrodactyly xenografts treated with BYL-719 or control on day 30 (right) (original magnification of upper images, ×100; down enlarged images, ×200). ***p < 0.001 by unpaired *t* test analysis. Scale bar: 100 μm.

We continued to examine whether BYL-719 could inhibit adipogenic differentiation in vivo. It was reported that BYL-719 had a favorable pharmacokinetic profile after oral administration^20^. We developed a mouse PDX model using adipose tissue of macrodactyly (Mac-AT) and dosed the mice with BYL-719 via intragastrical gavage (Fig. 5a). The volume increase of macrodactylous xenograft was dramatically inhibited by BYL-719 (Fig. 5b, e), as well as the weights of xenograft tissues (Fig. 5c). The mRNA expression levels of adipogenic markers including *PPAR γ* and *C/EBP α* were also decreased in BYL-719-treated adipose (Fig. 5d). Furthermore, immunohistochemical staining showed that p-AKT level was significantly lower in BYL-719-treated Mac-AT than control group. Moreover, the size of adipocytes was also dramatically decreased (Fig. 5f). The weight of mice had no difference between the two groups (Fig. S2D). We also found that the growth of Mac-AT was faster than adipose of polydactyly (Pol-AT; Fig. S2C) and BYL-719 had less effect on polydactyly adipose tissue compared to Mac-AT (Figs. S2A, B).

**Fig. 6:**
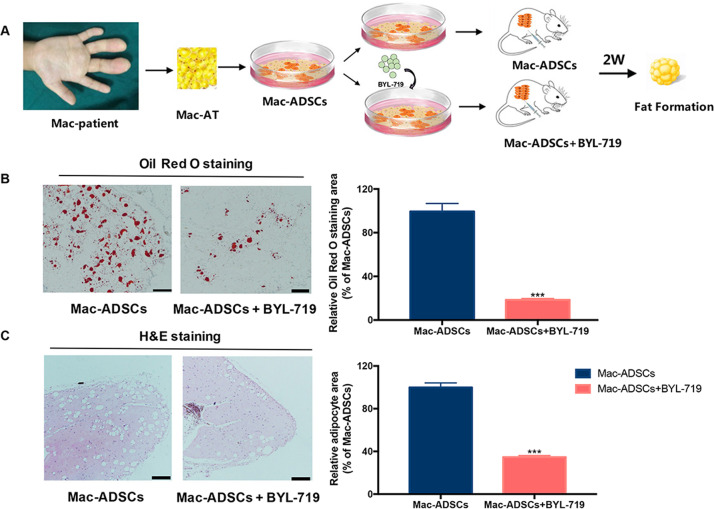
BYL-719 inhibited adipogenic differentiation of Mac-ADSCs in vivo. **a** Schematic description of the experimental procedure for PDC model. **b** Oil Red O staining of macrodactyly xenografts from BYL-719 treatment group and control group (*n* = 5). Quantitative assessment of Oil Red O staining was shown in right. **c** H&E staining of macrodactyly xenografts from BYL-719 treatment group and control group (*n* = 5). Quantitative assessment of adipocyte area was shown in right. Scale bar: 100 μm. ***p < 0.001 by unpaired *t* test.

To test the effect of BYL-719 on ADSCs-mediated adipose formation, we developed a PDC model of Mac-ADSCs (Fig.6a). The ADSCs-derived xenografts were harvested and analyzed by H&E and Oil Red O staining. Consistently, Oil Red O staining showed few positive areas in Mac-ADSCs treated with BYL-719 and H&E staining also revealed less fat area than the control group (Fig. 6b, c).

### E2F1 was a downstream target of PIK3CA in macrodactyly

**Fig. 7:**
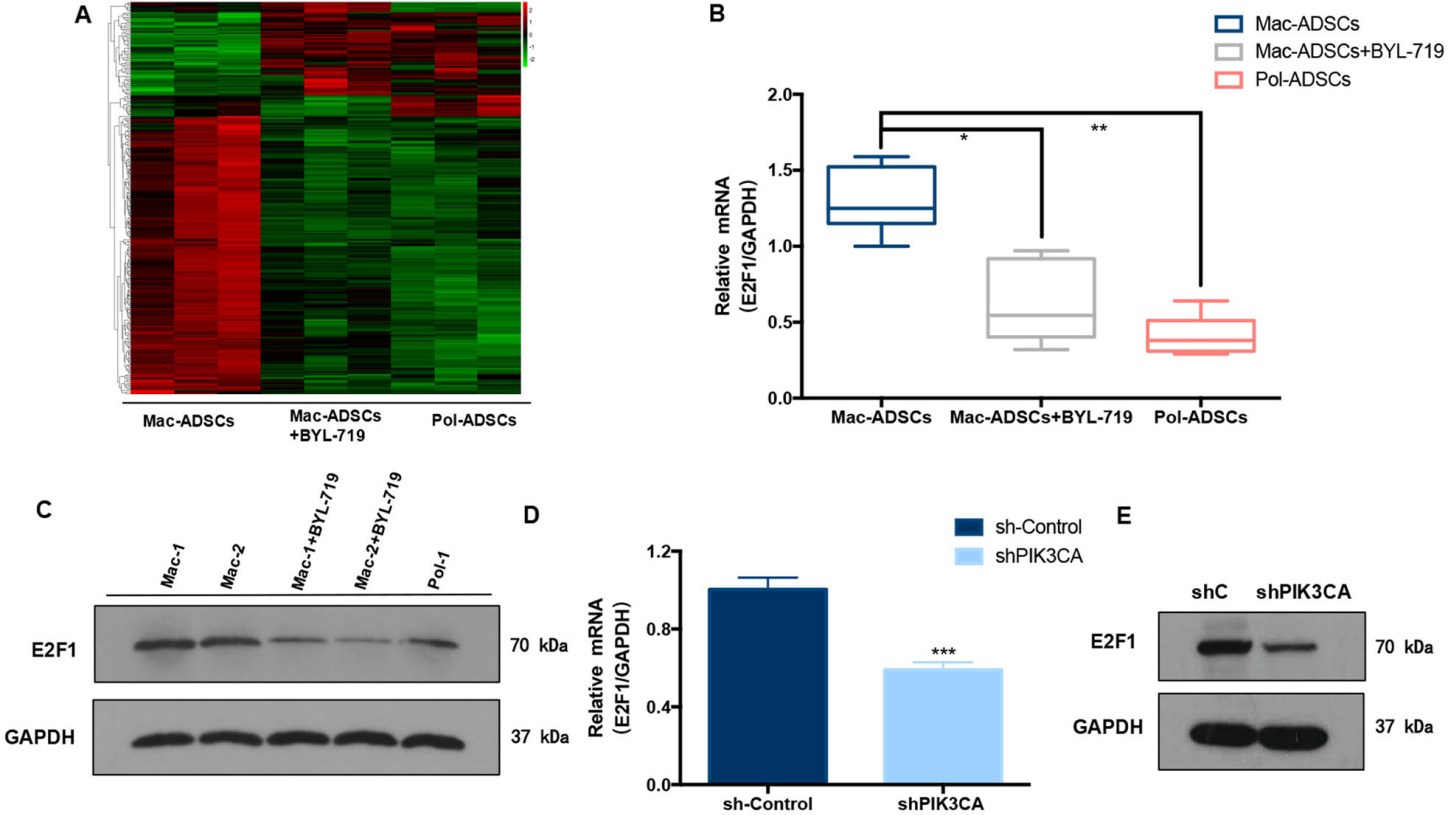
PIK3CA regulated E2F1 expression in Mac-ADSCs. **a**. Heat map representing color-coded expression levels of differentially expressed genes in Mac-ADSCs, Mac-ADSCs+BYL-719 (24 h,10 μM) and pol-ADSCs (three patients per group; GEO accession numbers: GSE151840). **b** The mRNA expression of E2F1 in Mac-ADSCs from six patients, Mac-ADSCs+BYL-719 (24 h, 10 μM) from six patients and pol-ADSCs from five patients respectively. **c**. Protein level of E2F1 in Mac-ADSCs, Mac-ADSCs+BYL-719 (24 h, 10 μM) and Pol-ADSCs respectively. **d**, **e**. The mRNA and protein levels of E2F1 in Mac-ADSCs with PIK3CA knockdown were detected by RT-qPCR and western blotting. Data are shown as the mean ± SD. *p < 0.05, **p < 0.01, ***p < 0.001 by ANOVA analysis or unpaired *t* test.

To identify the key downstream effectors of PIK3CA activation-mediated adipogenesis in Mac-ADSCs, we examined the transcriptome of Mac-ADSCs, BYL-719-treated Mac-ADSCs (24 h,10 μM), and Pol-ADSCs by RNA-sequencing (RNA-Seq) analysis. The heat map showed that 197 genes were significantly up-regulated and 71 genes were significantly down-regulated in Mac-ADSCs compared to the other two groups (fold change > 2, *p* value < 0.01; Fig. 7a). Our attention was drawn to E2F1, which encodes a transcription factor that has been shown to be closely associated with PI3K pathways^21–23^ and adipogenic differentiation^24–27^. E2F1 was highly expressed in the Mac-ADSCs and was decreased in the Pol-ADSCs and the BYL-719 treated Mac-ADSCs. The gene expression changes of E2F1 were further confirmed in Mac-ADSCs (*n* = 6), Mac-ADSCs+BYL-719 (*n* = 6), and Pol-ADSCs (*n* = 5) by RT-qPCR (Fig. 7b). The protein level of E2F1 was also decreased in BYL-719 treated Mac-ADSCs (*n* = 2) and Pol-ADSCs (*n* = 1) compared to Mac-ADSCs (*n* = 2) (Fig. 7c). Interestingly, E2F1 expression at both mRNA and protein levels was decreased following PIK3CA silencing (Fig. 7d, e). These results further supported E2F1 as a potential downstream target of PIK3CA.

### E2F1 regulated adipogenesis in macrodactyly

**Fig. 8:**
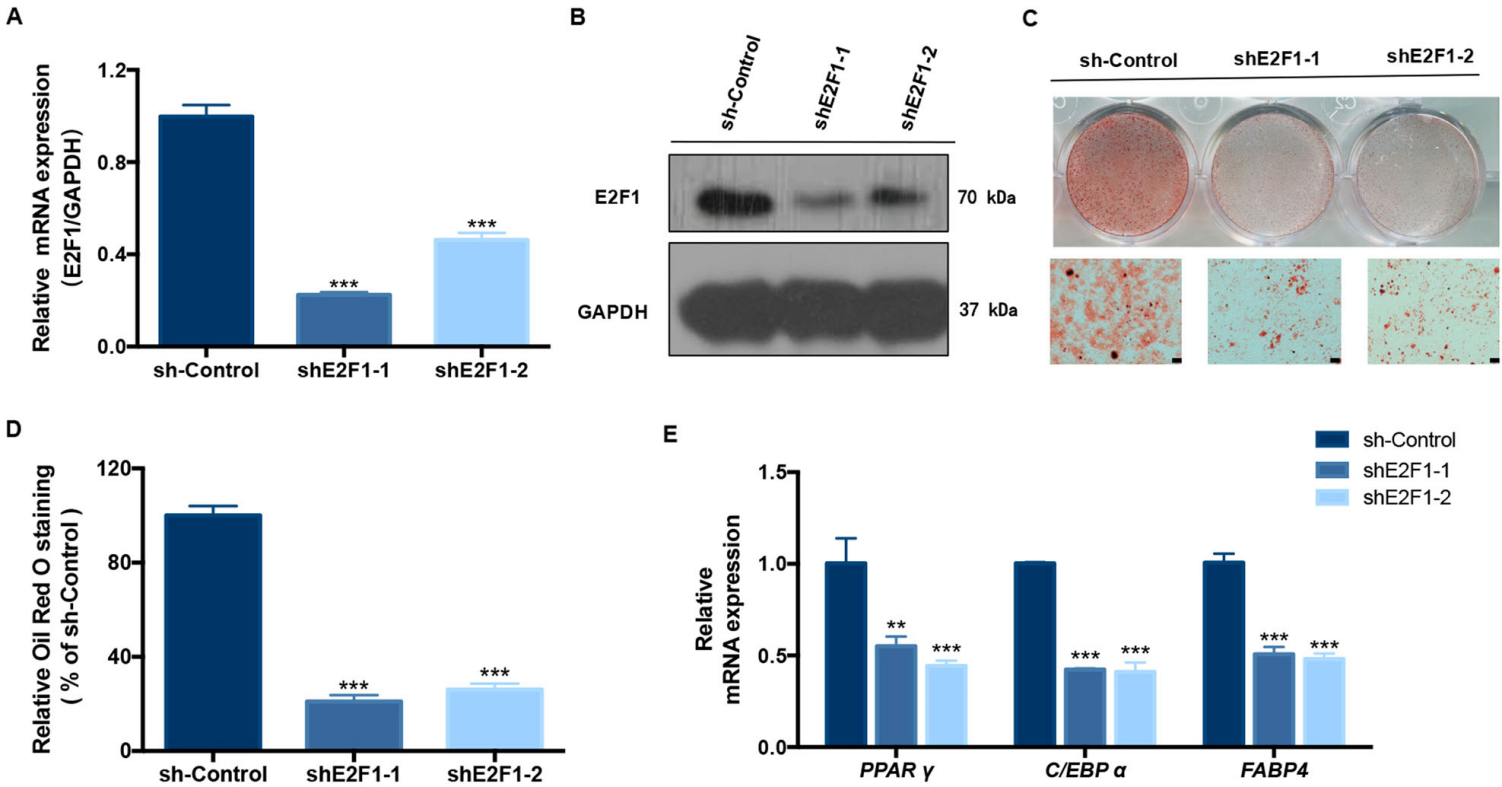
E2F1 mediated the effect of PIK3CA on adipogenesis in macrodactyly. **a** RT-qPCR verified the silencing of E2F1 after transfection of the shRNA into Mac-ADSCs with PIK3CA mutation. **b** Protein expression of E2F1 was measured by western blotting in Mac-ADSCs with PIK3CA mutation after E2F1 silencing. **c**, **d** Oil Red O staining and quantitation in E2F1 silenced Mac-ADSCs after adipogenic differentiation for 15 days. **e** The mRNA levels of adipogenic markers genes *PPAR γ*, *C/EBP α*, and *FABP4* in E2F1 silenced Mac-ADSCs were measured by RT-qPCR on day 3 of adipogenic differentiation. Data are shown as the mean ± SD. **p < 0.01, ***p < 0.001 by ANOVA analysis.

Several studies have reported a correlation between E2F1 expression and adipogenesis regulation^24–27^. To investigate whether E2F1 could regulate PIK3CA-induced hyper-adipogenesis in Mac-ADSCs, we transfected Mac-ADSCs with shE2F1 to silence E2F1 expression (Fig. 8a, b). The results revealed that knockdown of E2F1 significantly reduced adipogenesis of Mac-ADSCs (Fig. 8c, d). In addition, the mRNA levels of adipogenesis regulating genes, *PPAR γ*, *C/EBP α*, and *FABP4*, were significantly decreased (Fig. 8e). These data indicated that the effect of PIK3CA on adipogenesis in Mac-ADSCs could be regulated by E2F1.

## Discussion

Macrodactyly is one of the most challenging congenital limb anomalies because current surgical treatments such as debulking often lead to relapse. In recent years, PIK3CA mutation and the resulting activation of PI3K/AKT signaling pathway has been considered as the etiology for macrodactyly, and macrodactyly has been categorized into PIK3CA-related overgrowth spectrum (PROS)^2,12^. Excessive accumulation of adipose tissue is a striking feature in macrodactyly and PIK3CA mutations were detected in adipose tissues from macrodactyly patients^11,28^. However, there is a lack of research exploring the effect and mechanism of PIK3CA activating mutation on adipogenesis in macrodactyly. In this study, we discovered that the PIK3CA activating mutation promoted adipogenesis of ADSCs in macrodactyly, and that this effect could be exerted by the up-regulation of E2F1.

PIK3CA activation has been reported in many solid tumors. The mutation H1047R of PIK3CA resides in the C-terminal region of the kinase domain and has been proven to be an activating or gain-of-function mutation^29,30^. PI3K/AKT signaling pathway was known to promote lipid biosynthesis and inhibit lipolysis by directly regulating protein kinase A (PKA)^31^. In addition, Elise et al^32^. and Even D^16^ reported that activation of adipogenesis in adipocyte precursors was dependent on PI3K/AKT pathway and down-regulation of the PI3K pathway inhibited the adipogenesis in 3T3-L1 and primary mesenchymal stem cells^33^. In our study, PIK3CA mutation (H1047R) was detected in all Mac-ADSCs patients, resulting in hyper-activation of PI3K/AKT pathway compared to Pol-ADSCs. In addition, Mac-ADSCs exhibited enhanced Oil Red O stained cells as compared to Pol-ADSCs. The results were further supported by the reduction of adipogenesis in PIK3CA silenced Mac-ADSCs. Clinically, macrodactyly presents a wide array of clinical phenotypes, with the location, rate and extent of overgrowth varying greatly among individual patients. Hot spot mutations such as E542K and H1047R were associated with significantly elevated biological and biochemical activities comparing to the rare mutations^12^. Different mutations could lead to different degrees of pathway activation and overgrowth severity. The exact correlation between phenotype and genotype is worth further investigation.

Since small molecule inhibitors have been approved to block the PI3K/AKT/mTOR pathway activated aberrantly by the PIK3CA mutation in solid tumors, targeted therapy could become a promising alternative remedy for PROS. Loconte et al.^34^ reported that treating the dermal fibroblasts from PROS patients with the PI3K inhibitors wortmannin and LY294002 in vitro resulted in a significant reduction of cell proliferation. Suzuki et al.^35^ found that rapamycin and NVP-BEZ235 inhibited the cell growth of both dermal fibroblast lines from PROS patient and the control normal cells. Parker et al.^36^ treated thirty-nine PROS patients with mTOR inhibitor sirolimus in vivo for 26 weeks and revealed that low-dose sirolimus could modestly reduce overgrowth, while that the side-effect profile was significant with 72% adverse event and 37% severe adverse event. BYL-719, a specific PI3Kα inhibitor approved for breast cancer treatment^37^, was reported to alleviate symptoms including heart failure, hemihypertrophy and scoliosis for all 19 PROS patients and no substantial side effects were observed^38^. In addition, BYL-719 demonstrated a tolerable safety margin and encouraging efficacy in patients with PIK3CA-altered solid tumors^39^.

Considering the critical roles of PIK3CA hyper-activation in adipogenesis, PI3K targeted therapy provides a good opportunity to inhibit the overproduction of adipose in macrodactyly. In this study, we investigated the effect of BYL-719 on macrodactyly for the first time and demonstrated that the use of BYL-719 dramatically inhibited adipogenic differentiation of Mac-ADSCs in vitro. The PDX model has been adopted as a major tool for evaluating preclinical drugs in vivo. We established a PDX model with macrodactyly xenograft and revealed that BYL-719 treatment significantly decreased p-AKT level and adipose tissue growth. It was observed that BYL-719 preferentially suppressed macrodactyly tissue than polydactyly tissue, which was consistent with the study by Fritsch et al.^40^, who demonstrated that cells lines carrying PIK3CA mutation were more likely to be responsive to BYL-719 than the wild-type control. The inhibitory effect was also observed in a PDC model in which Mac-ADSCs were transplanted subcutaneously after adipogenic induction in the medium supplemented with BYL-719. Notably, all animals tolerated BYL-719 administration well. Taken together, these findings indicated that BYL-719 is potentially a safe and effective drug candidate for inhibiting adipogenesis in macrodactyly.

To unveil the underlying molecular mechanism of PIK3CA activation-mediated adipogenesis in Mac-ADSCs, RNA-Seq was performed to look into downstream effectors of PIK3CA activating mutation. E2F1 was identified as one of the differentially expressed genes which was highly expressed in Mac-ADSCs but was decreased in Pol-ADSCs or by BYL-719 treatment. Studies have shown the connections between E2F1 and PI3K/AKT pathway. The regulation of E2F1 expression by TopBP1 is dependent on the PI3K/AKT signaling pathway^23^ and PI3K/AKT pathway could control the E2F1 proliferative or apoptotic function by regulating E2F1 targeted genes^21,22^. Moreover, E2F1 had been reported to induce *PPAR γ* transcription in 3T3-L1 cells^24,41^ and served as an important link between preadiocyte proliferation and differentiation in duck^27^. We further investigated E2F1 due to its involvement in adipogenesis regulation. In our study, E2F1 was highly expressed in the Mac-ADSCs group and knockdown of E2F1 was accompanied with decreased adipogenesis in Mac-ADSCs. The smaller size of adipocytes observed in the PDX model after BYL-719 treatment was possibly correlated with the inhibition of E2F1, since it was reported that decreased adipocyte size was associated with down-regulation of E2F1 expression^42^. Thus, we hypothesize that the the molecular mechanism of adipogenesis in macrodactyly is regulated by E2F1 upon activation of PI3K/AKT pathway.

There are two noteworthy points in this study. The first one is the selection of a control group. Theoretically adipose tissue from the self-normal digit is the best control group, however it is not ethically possible. Moreover, it is difficult to obtain adipose tissue from an age-matched allogeneic finger. Specimen from polydactyly has been used as control group in reported studies on congenital hand deformities^43,44^. Therefore, after careful consideration, we chose the adipose tissue and ADSCs from polydactyly allogenically as a control group in this study. The second point is regarding the downstream effectors of PIK3CA. E2F1 was identified after RNA sequencing and selected based on its connections with PI3K/AKT pathway and adipogenesis reported in literature. However, we cannot rule out the possibility that other unknown targets beyond E2F1 exist and mediate adipogenesis upon activation of PIK3CA. Further studies are planned to explore the additional mechanisms of adipogenesis in macrodactyly.

PI3K/AKT signaling pathway is critical for normal metabolism, and its imbalance leads to the development of obesity and type 2 diabetes mellitus^31^. Evidence revealed BYL-719 was sufficient to increase energy expenditure and reduce obesity in mice^45^. Considering this, further study should be done focusing on the glucose and lipid metabolism of adipose tissue in macrodactyly. Since somatic mutation of PIK3CA has been identified in other affected tissue types such as bone, muscle, skin, and nerve of macrodactyly patients^11^, BYL-719 could potentially inhibit the overgrowth of these tissues with PIK3CA gain-of-function mutations. In fact, our group also found activating PIK3CA mutation promoted osteogenesis of bone marrow mesenchymal stem cells in macrodactyly and BYL-719 treatment significantly reduced osteogenic differentiation (data no shown). More importantly, considering that a large spectrum of diseases harbor the PIK3CA gain-of-function mutations gene, PIK3CA inhibitors such as BYL-719 could become an effective therapeutic candidate for PROS including megalencephaly capillary malformation (MCAP), CLOVES, muscle hemihypertropy, and epidermal nevi.

This study demonstrated for the first time that PIK3CA activating mutation promoted excessive adipogenesis of Mac-ADSCs via up-regulation of E2F1, elucidating a possible mechanism for lipid accumulation in macrodactyly. Furthermore, BYL-719, a PIK3CA inhibitor, was applied to research in macrodactyly and demonstrated its potential therapeutic effect. The study lays the basis for further investigation and potential clinical application of BYL-719 in treating macrodactyly.

## Materials and methods

### Patient information

Surgically amputated digits of three macrodactyly patients and three polydactyly patients were obtained from Department of Plastic and Reconstructive Surgery, Shanghai Ninth People’s Hospital. All protocols involving human subjects were reviewed and approved by the Institutional Review Board of Shanghai Ninth People’s Hospital (No: 201580). All procedures were carried out in accordance with guidelines set forth by Declaration of Helsinki. Written informed consent was obtained. The detailed clinical information was listed in Table S1.

### Isolation and culture of human ADSCs

The Isolation and culture of human ADSCs were performed as previously described^7^. The passages of all ADSCs used in this study were below five.

### Cell surface markers staining and flow cytometry

Cell surface markers staining and flow cytometry were performed as previously described^7^.

### DNA isolation and sequencing

Genomic DNA was extracted from ADSCs using QIAamp DNA Mini Kit (Qiagen, Valencia, CA, USA). DNA concentrations were determined by NanoDrop spectrophotometer (Thermo Fisher Scientific, MA, USA). Conventional PCR was performed using a Veriti thermocycler (Applied Biosystems, Thermo Fisher Scientific MA, USA). Amplified PCR products were analyzed by Tsingke (Shanghai, China) for Sanger sequencing.

### Adipogenic differentiation

Adipogenic differentiation was performed as described previously^7^. The cells were incubated according to according to the manufacturer’s instruction (Cyagen, CA, USA). Quantitative analysis of the Oil Red O stain was performed by dissolving the stained lipid droplets with isopropanol followed by absorbance reading at 520 nm. Relative Oil Red O stain was presented as percentage with the correspondent control as 100%.

### Western blotting

Total protein was extracted from ADSCs using RIPA Lysis Buffer (Beyotime Institute of Biotechnology). Protein lysates were resolved by sodium dodecyl sulfate-polyacrylamide gel electrophoresis (SDS-PAGE), followed by protein transfer onto PVDF (polyvinylidene difluoride) membranes (Millipore). After being blocked with 5% nonfat dry milk or bovine serum albumin (BSA) for 30 min, PVDF membrane was incubated with primary antibodies overnight. The blots were washed with TBST and incubated with homologous HRP-conjugated secondary antibodies (Jackson) for 45 min. The proteins were visualized using ECL Detection System (Thermo Scientific) and quantitated by Image-Pro Plus 6.0 software. The following primary antibodies were used: PIK3CA (Abcam, Cat#ab40776), p-AKT (Cell Signaling Technology, Cat#13038), AKT (Cell Signaling Technology, Cat #4691), p-mTOR (Cell Signaling Technology, Cat #5536), mTOR (Cell Signaling Technology, Cat#2983), p-s6 (Cell Signaling Technology, Cat#4858), s6 (Cell Signaling Technology, Cat#2317), E2F1 (abcam, Cat#ab179445), and GAPDH (Cell Signaling Technology, Cat#5174).

### RNA extraction and RT-qPCR

RNA extraction, reverse transcription, and qPCR were performed as previously described^7^. Adipogenic markers were examined at day 3 of adipogenic differentiation. E2F1 in BYL-719-treated Mac-ADSCs was examined 24 h after BYL-719 treatment (10 μM). Specific primers used in this study for *PPAR γ*, *C/EBP α*, *FABP4*, *PIK3CA*, *E2F1*, and *GAPDH* were listed in Table S2. Relative gene expression was examined using the 2^−ΔΔct^ method.

### Lentiviral shRNA transfection

The lentiviral vector containing short hairpin (sh) RNAs targeting human PIK3CA and E2F1 were purchased from Zuorun Biotechnology (Shanghai,China). Lentivirus was transfected in HEK293T cells and the supernatant of the medium was harvested after 48 h. ADSCs were seeded into a 6-well plate (3 × 10^5^/well) and settled overnight. ADSCs were infected with virus (MOI = 10) for 12 h in the presence of polybrene (8 μg/ml) and maintained in regular medium for 48 h. Transduced cells were selected in the presence of 4 μg/ml puromycin for 3 days. The expression of PIK3CA and E2F1 was analyzed by RT-qPCR. The primers used for shRNA and control were listed in Table S2.

### Mouse PDX and PDC model

Patient-derived xenograft model (PDX model): all animal experiments were approved by Shanghai Ninth People’s Hospital Animal Experimentation Ethics Committee. Six-week-old female nude mice were obtained from Shanghai SLAC Laboratory Animal Company. The adipose tissue from macrodactyly patient was cut into 2 × 2 × 3 mm^3^ pieces. The mice were anesthetized by chloral hydrate (0.004 mL/g). Horizontal incisions of 5 mm were made on dorsum of mice to create enough subcutaneous space and fragments of adipose tissue were implanted. The animal dosing started when the xenograft grew to ~25 mm^3^. For adipose tissue of macrodactyly (Mac-AT), two groups of mice (*n* = 6 per group) were assigned randomly to receive either methylcellulose 0.5% (control group) or BYL-719 (50 mg/kg) by intragastrical gavage daily. The same procedure was performed for implanted polydactyly adipose tissue (Pol-AT). The adipose volume was measured by Vernier calipers and calculated using the formula of (Length × Width^2^)/2. Mice were killed on Day 30 by CO_2_ overdose followed by cervical dislocation. The adipose tissues were triaged for photograph, weighing, histology, and molecular analysis. Relative tissue volume was assessed by calculating the ratio of adipose volume at measurement day over the initial volume of Day 1 (start of treatment).

Patient-derived ADSCs model (PDC model)*:* Mac-ADSCs (P2) were induced for adipogenic differentiation by adipogenic medium with or without BYL-719 (10 μM) for 3 days. The differentiated Mac-ADSCs cells (1×10^6^) were suspended in 20 μl of 1:1 PBS and Matrigel^TM^ (BD Bioscience) and subcutaneously injected into the right flank of 6-week-old female nude mice (*n* = 5 per group). After 2 weeks, these transplants were collected for Oil Red O and H&E staining. Five images in the slides were randomly selected from each group and captured under microscopy. The area of new adipocyte in each image was marked using Image-Pro Plus 6.0 software and the percentage of positive staining was calculated using untreated Mac-ADSCs group as a control (100%).

### Immunohistochemistry

Immunohistochemistry was performed as described previously^7^. Primary antibody was p-AKT (T308) (Abcam, Cat #ab38449) at 1:200 dilution.

### RNA-sequencing analysis

Total RNA of Mac-ADSCs, Mac-ADSCs treated with BYL-719 treated (24 h, 10 μM), and Pol-ADSCs derived from three patients was purified using RNeasy mini kit (Qiangen, Germany). Complementary DNA library preparation and sequencing were performed according to Illumina standard protocol. Differential expression analysis was performed using edgeR package. An absolute log2 fold change >1 and *q*-value < 0.05, set as the criteria for significantly modulated genes, were retained for further analysis (GEO accession numbers: GSE151840).

### Statistical analysis

Results were expressed as mean ± SD. All experiments were performed at least three times independently. One-way ANOVA analysis or unpaired *t* test was used to determine if there are any statistically significant differences between the experimental groups. A *p-*value <0.05 was considered statistically significant (*p < 0.05, **p < 0.01, and ***p < 0.001). All statistical analyses were performed with Graph Pad Prism 6 software (Graph Pad Software).

## Supplementary information

Supplementary Information

Table s1

Table s2

Supplementary Figure 1

Supplementary Figure 2

Supplementary Figure 3
