# Supplementary material for: Activating PIK3CA mutation promotes adipogenesis of adipose-derived stem cells in macrodactyly via up-regulation of E2F1
Source: Cell Death Dis. 2020 Jul 30;11(7):600. doi: 10.1038/s41419-020-02806-1 (PMC7393369; doi:10.1038/s41419-020-02806-1)
Supplement: Supplementary file 1 — Supplementary Information [file 41419_2020_2806_MOESM1_ESM.docx]

**Supplementary Information**

**Fig.S1 Cell morphology and surface markers of Mac-ADSCs and Pol-ADSCs.** A, B. Cell morphology of Mac-ADSCs and Pol-ADSCs were shown. Scale bar: 100 μm C, D. ADSC-specific markers CD34, CD45 and CD29 in Mac-ADSCs and Pol-ADSCs were examined by flow cytometry.

**Fig.S2 The effect of BYL-719 on adipose formation of adipose tissues in macrodactyly and polydactyly *in vivo*.** A. Weight from BYL-719 treated and control Mac-AT and Pol-AT on day 30 (n=6). B. Adipose volume from BYL-719 treated and control Mac-AT and Pol-AT on day 30 (n=6). C. The curve of relative adipose volume of BYL-719 treated and control Mac-AT and Pol-AT during the 30 days of treatment (n=6). D. Body weight of mice receiving BYL-719 and control during the 30 days of treatment (n=6).

**Fig.S3 A schematic view of activating PIK3CA mutation promotes adipogenesis of adipose-derived stem cells in macrodactyly.** Gain-of-function mutation of PIK3CA enhanced activity of PI3K/AKT pathway and increased potential of adipogenic differentiation in Mac-ADSCs. This effect was exerted by the up-regulation of E2F1. BYL-719 was indicated as a potential therapeutic agent for macrodactyly treatment.
