# Supplementary material for: Activating PIK3CA mutation promotes adipogenesis of adipose-derived stem cells in macrodactyly via up-regulation of E2F1
Source: Cell Death Dis. 2020 Jul 30;11(7):600. doi: 10.1038/s41419-020-02806-1 (PMC7393369; doi:10.1038/s41419-020-02806-1)
Supplement: Supplementary file 2 — Table s1 [file 41419_2020_2806_MOESM2_ESM.docx]

| **Table S1. Clinical information of patients with macradactyly and polydactyly** | | | |
| --- | --- | --- | --- |
| Patient Number | Sex | Age | Diagnosis |
| NO.1 | male | 11y | Macrodactyly（right hand） |
| NO.2 | female | 4y | Macrodactyly（left hand） |
| NO.3 | male | 1y | Macrodactyly（left hand） |
| NO.4 | male | 1y | Macrodactyly（left foot） |
| NO.5 | male | 8y | Macrodactyly（right hand） |
| NO.6 | female | 1y | Macrodactyly（left foot） |
| NO.7 | male | 1y | polydactyly（right hand） |
| NO.8 | male | 1y | polydactyly（right and left hands） |
| NO.9 | female | 13m | polydactyly（right hand） |
| NO.10 | female | 10m | polydactyly（right hand） |
| NO.11 | female | 1y | polydactyly（left hand） |
