# Supplementary material for: Activating PIK3CA mutation promotes adipogenesis of adipose-derived stem cells in macrodactyly via up-regulation of E2F1
Source: Cell Death Dis. 2020 Jul 30;11(7):600. doi: 10.1038/s41419-020-02806-1 (PMC7393369; doi:10.1038/s41419-020-02806-1)
Supplement: Supplementary file 3 — Table s2 [file 41419_2020_2806_MOESM3_ESM.docx]

| **Table S2. Prime sequence for RT-PCR and shRNA** | |
| --- | --- |
| Genes | Primer sequence |
| E2F1 | F:ATCACCAACGTCCTTGAGGG |
|  | R:GTCCTGACACGTCACGTAGG |
| PPAR γ | F:TGTCTCATAATGCCATCAGGTTTG |
|  | R:GATAACGAATGGTGATTTGTCTGTT |
| C/EBP α | F:GGACCCTCAGCCTTGTTTGT |
|  | R:TGGTGGTTTAGCAGAGACGC |
| FABP4 | F:ACCAGGAAAGTGGCTGGCAT |
|  | R:CAGGTCAACGTCCCTTGGCT |
| PIK3CA | F:TCGCCTCATAGCAGAGCAAT |
|  | R:AGGACAACAACATGCTCCGA |
| GAPDH | F:GGAGCGAGATCCCTCCAAAAT |
|  | R:GGCTGTTGTCATACTTCTCATGG |
| shControl | 5’-CCTAAGGTTAAGTCGCCCTCG-3’ |
| shPIK3CA-1 | 5’-GCTTGAAGAGTGTCGAATTAT-3’ |
| shPIK3CA-2 | 5’-AGAATATCAGGGCAAGTATAT-3’ |
| shE2F1-1 | 5′-CGTGGACTCTTCGGAGAACTT-3′ |
| shE2F1-2 | 5′-CGCTATGAGACCTCACTGAAT-3′ |
