# Supplementary figures and images for: Activating PIK3CA mutation promotes adipogenesis of adipose-derived stem cells in macrodactyly via up-regulation of E2F1
Source: Cell Death Dis. 2020 Jul 30;11(7):600. doi: 10.1038/s41419-020-02806-1 (PMC7393369; doi:10.1038/s41419-020-02806-1)

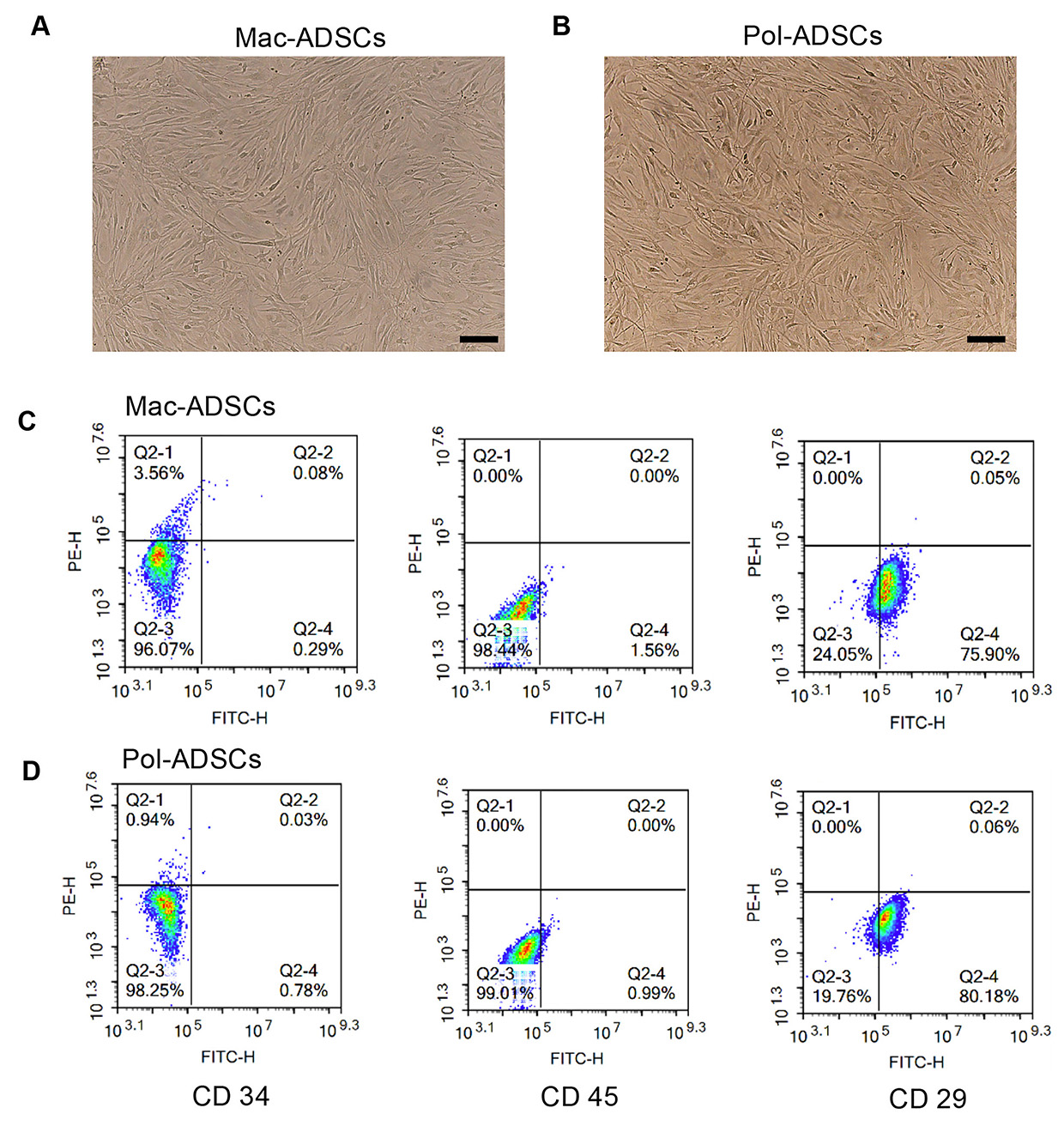

Supplement: Supplementary file 4 — Supplementary Figure 1 [file 41419_2020_2806_MOESM4_ESM.png]

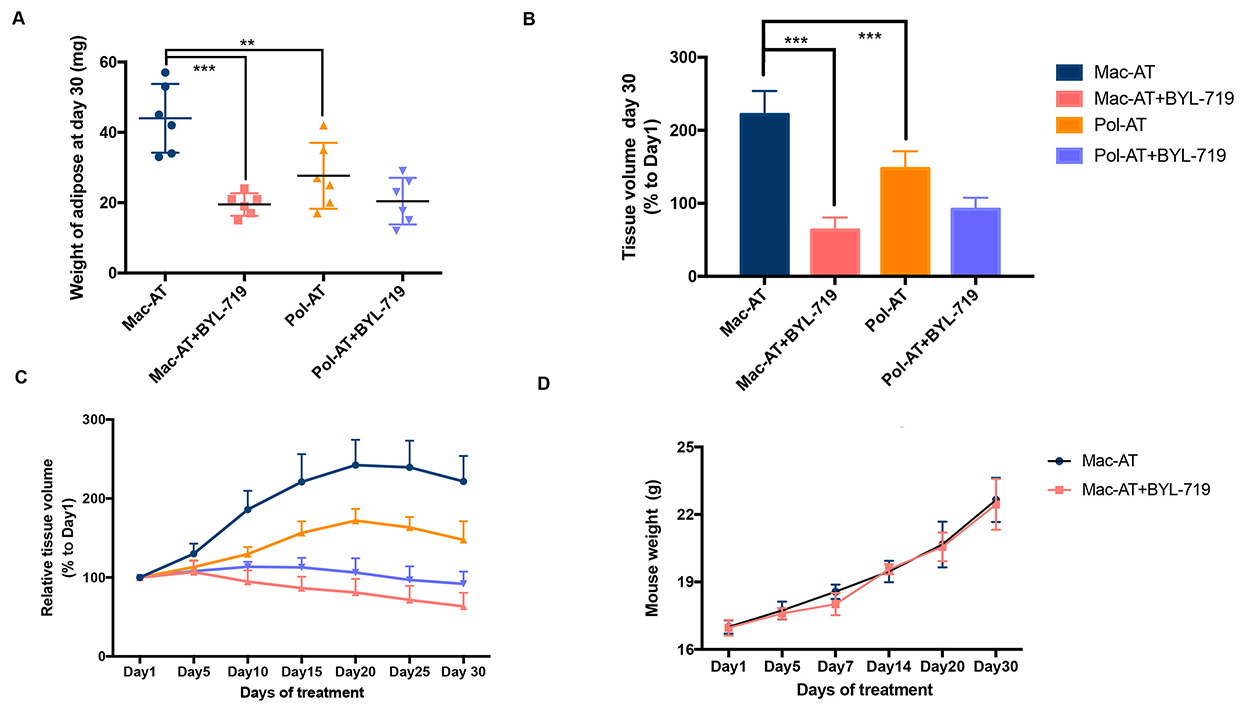

Supplement: Supplementary file 5 — Supplementary Figure 2 [file 41419_2020_2806_MOESM5_ESM.png]

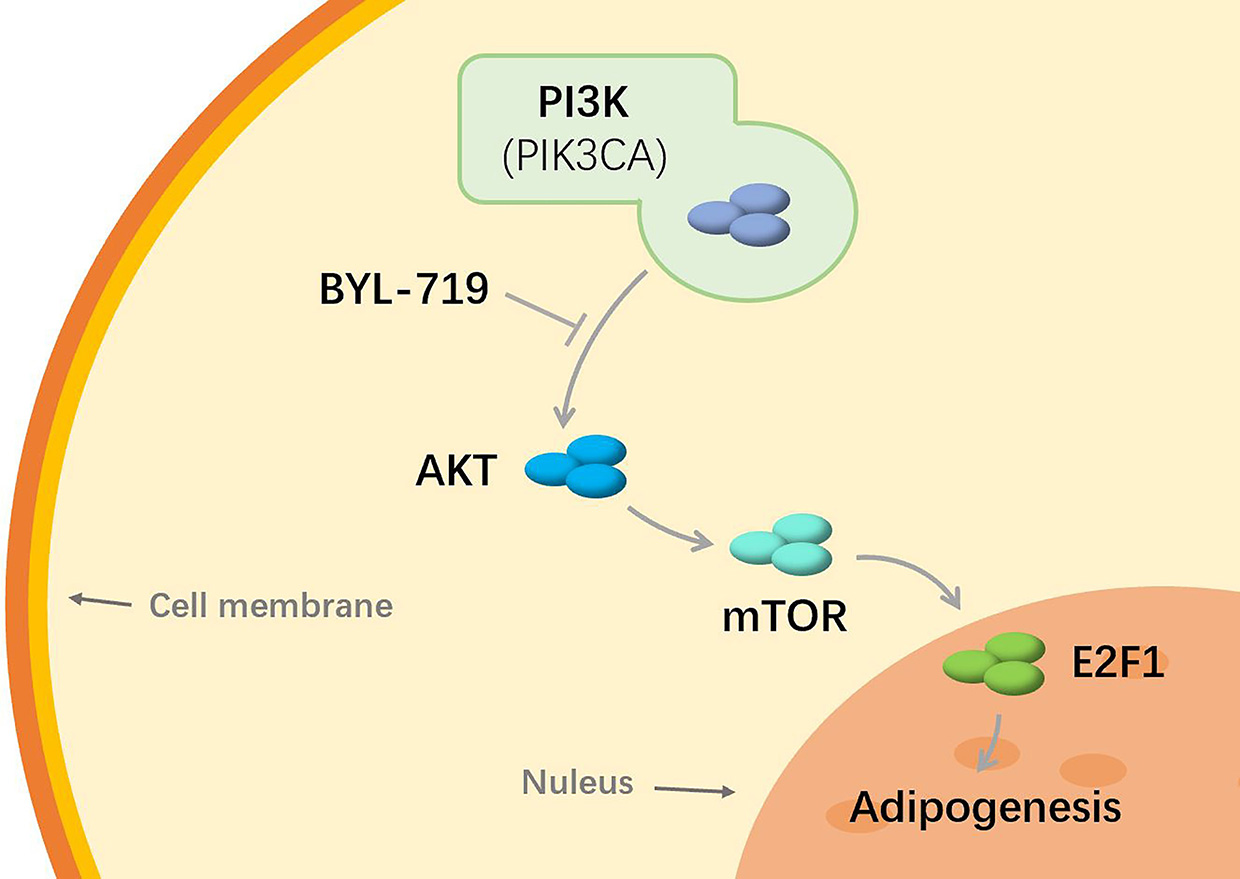

Supplement: Supplementary file 6 — Supplementary Figure 3 [file 41419_2020_2806_MOESM6_ESM.png]
